# The Training of an Asylum Matron

**Published:** 1918-07-06

**Authors:** George M. Robertson

**Affiliations:** Physician-Superintendent of the Royal Edinburgh Asylum.


### THE EDITOR'S LETTER-BOX.

### The Training of an Asylum Matron.

*To the Editor of* The Hospital.

Sir,—I was much interested in Captain Kirkland-Whittaker's remarks at the annual meeting of the Asylum Workers' Association (the *Asylum News*, p. 18) on the promotion of mental nurses to the posts of assistant matron and matron, and from the applause they received it would appear that the sentiments were approved of by the whole meeting. It is interesting to record that he repeats in England the views which were expressed in Scotland by Dr. Yellowlees, of Gartnavel (the father of the Medico-Psychological Association), so long ago as 1898. While agreeing with Captain Kirkland-Whittaker that the matron of an asylum ought to be fully qualified in her profession and hold both the certificates of hospital nursing and of proficiency in mental nursing, he thought the best matrons were those who had first been mental nurses and had subsequently completed their training in the wards of a general hospital. The asylum, he eloquently said, was their "first love," and their interest in work of this kind of institution would be greater.

Dr. Yellowlees' remarks were made during a discussion on the training of hospital nurses in mental work for the purpose of fitting them to become matrons of asylums. A considerable number since 1880 had been appointed matrons of asylums in Scotland, and owing to their want of training, with indifferent success in many cases. I thought this defect should be rectified, and I induced the first hospital nurses to enter the wards of an asylum in the year 1896. The prestige of the Asylum Service was then so low that it took nearly a year before I could get a single candidate. Two others came shortly afterwards. All three became matrons of asylums within three years, and after that candidates became numerous. The full double training is, of course, a tremendous advantage to any applicant for a matron's post. No person should, however, be appointed matron of an asylum, if it can be avoided, who does not hold the certificate of proficiency in mental nursing, and the training which was instituted in 1896 for hospital nurses and adopted in many other asylums since then obviates the necessity of doing this.

In answer to Dr. Yellowlees I replied that enterprising and intelligent mental nurses, meeting hospital nurses in the wards of asylums, would be induced to complete their training in hospitals, and would in their turn be available for matrons' posts. Also that the status of asylum nurses would be improved by hospital nurses working in the wards of asylums. My surmises both proved correct. Scores of my nurses have taken their hospital training. I fancy this practice is more prevalent in Scotland than in England, as our asylums have become more hospitalised and we employ more hospital nurses in them. The result of this is, that many mental nurses who have completed their double training in hospitals are afterwards appointed assistant matrons, and some do ultimately become matrons of asylums. Lately, I think, four out of five assistant matrons at the West House of the Morningside Royal Asylum had begun their career as mental nurses. During the last three years three at least, if not more, of my former assistant matrons, who started as mental nurses, have been appointed matrons of English asylums. Another, I believe, is on a short leet. More mental nurses may, therefore, be appointed matrons than Captain Kirkland-Whittaker suspects, but they usually have the double training which we all think so desirable. In any case good mental nurses are now coming into their own in this respect, as I predicted twenty years ago they would, and the status of mental nurses is much higher now than then.

Hard lines still occur, as when a faithful experienced mental nurse is passed over for a younger woman who holds both certificates. Some weight must of course be attached to the possession of the second certificate, and the interests of the patients and the institution must come first. One cannot fail to sympathise with these older officials, not so much, however, with the younger generation of mental nurses. Those of them who are enterprising and ambitious should know by this time that if they aspire to the higher posts they must complete their training in a general hospital. If they do this, there are many I know of, like Dr. Yellowlees, who will give them a preference when opposed by candidates who are equally qualified but whose "first love" has not been the asylum.

I do not think there is any difficulty such as Captain Kirkland-Whittaker suggests in a mental nurse completing her training in a general hospital. My experience, which is not exceeded by anyone, is opposed to this. She must, of course, resign her asylum post after obtaining her certificate, which she can do by giving a month's notice. She will find she will have less difficulty than the untrained woman in entering a hospital, as the certificate of the Association which she possesses, I am proud to say, is held in high esteem. The matron of the hospital knows that she is not a raw untrained ordinary probationer. If she has done good service in the asylum, the superintendent and the matron will help her to enter a hospital.

I think it distinctly hard that one year should not be deducted from the three required for hospital training, in virtue of her mental certificate, as is done when a hospital nurse enters for the mental certificate. This point has already been brought by the Medico-Psychological Association to the notice of the College of Nursing, and the favour will no doubt be obtained in time. It was several years before the Medico-Psychological Association itself granted the favour to hospital nurses. As I was the first to train hospital nurses in asylums, I naturally proposed at a meeting that this favour should be accorded them, but I underwent the trying experience of not finding anyone to second my proposal. Several years afterwards, at a large meeting in London at which I was present, Dr. Mercier made a similar proposal, and he not only found a seconder but his motion was enthusiastically carried without a dissentient voice. His argument may have been as lucid, interesting, and convincing on that occasion as his speech was at the annual meeting, and the times may have been ripe.—I am, etc.,

George M. Robertson, M.D., F.R.C.P.Ed.,
Physician-Superintendent of the
Royal Edinburgh Asylum.

June 25, 1918.

